# Remedies and Their Uses

**Published:** 1906-06-30

**Authors:** 


### Remedies and their Uses.

### Oxaphor and Camphor.

Oxaphor is a 50 per cent. solution of oxycamphor in alcohol, and has been recommended as a respiratory stimulant. Chemically it is camphor in which one hydrogen atom is replaced by hydroxyl (OH). It is prepared synthetically by Meister, Lucius, and Brüning, of Hoechst. It is a white crystalline powder, not very soluble in water, and liable to decompose after a time on exposure to the air. The alcoholic solution in which the drug is sold is, however, sufficiently stable. It has little effect on healthy individuals, even in large doses (10 grams), but in animals it has been shown by Heintz to act specially as a stimulant to the respiratory centres. In pathological conditions in man it has been found useful in all kinds of dyspnœa, whether arising from pulmonary, cardiac, or toxic sources. Thus A. Ehrlick (Zentralb. f.d. gesamte Therap., 1899, 1 and 2) has used it in 32 cases, and reports that it seldom fails to improve the subjective sensations of the patient, and that the best results are found in pulmonary conditions, such as tuberculosis and in cardiac dyspnœa. It has been observed to reduce the respiration rate from 56 to 23 in the course of one hour. Besides its specific stimulating effect on the respiratory centre oxycamphor also acts as a general sedative. It can also be employed in renal asthma, in anæmic conditions, and in nephritis with failing respiration. It appears to be specially valuable in the paroxysms of asthma. Hirschfelder (Occ. Med. Times, 1900, 12, 377) reports a case in which after .8 gram (12 grains) of the solid drug the symptoms were diminished in ten minutes, and shortly afterwards completely disappeared. No observers have reported any bad after-effects from the employment of oxycamphor. As to dosage, 5 to 15 grains, with a total of 30 to 60 grains in the 24 hours are the usual amounts given. Ehrlich does not think that doses under 15 grains are likely to be effective. His average dose was between 15 and 30 grains, with a maximum of 45 grains. Schriener (Therap. Monartsh, June 1903) gives, in cases of whooping-cough in children, 1½ to 3 grains for each year of age, from thrice daily to every three hours.

In prescribing oxaphor, which is a 50 per cent. solution, these doses must, of course, be translated into fluid measure and doubled. Thus 15 grains of oxycamphor will equal 30 minims of oxaphor. For a rapid effect it should be given on a fasting stomach. The following formulæ will be found useful, as the taste is objected to by some patients:

| | | |
|---|---|---|
| ℞ | Oxaphori ... ... ... ... | gr. 10-15 |
| | Syr. aurant ... ... ... ... | ʒj. |
| | Sp. vini gall. ... ... ... ... | ♏ 40 |
| | Aq. menth. pip. ad ... ... ... | ℥j. |

| | | |
|---|---|---|
| ℞ | Oxaphor ... ... ... ... | gr. 15 |
| | Sp. vini rect. ... ... ... ... | ♏ 30 |
| | Mist. amygdal. ad ... ... ... | ℥j. |

Camphor.—Camphor itself is probably but little used in this country except as a domestic remedy for the common cold, in which case Rubini's Tincture is generally employed. In France, however, it is more commonly given, mainly in respiratory affections. Thus Scheffler ("La Médicine Moderne," March 21, 1906) has given camphor in twenty-four cases of pneumonia during the last three years with two deaths. In one of these death was due to Bright's disease, and in the other the patient, who was eighty-two years of age, succumbed during convalescence to an attack of diarrhœa. In the remaining cases the disease ran a favourable course, crisis occurring in eighteen before the seventh day, and in only one was there any complication (serous pleurisy). The effect of the drug is seen in the reduction of temperature (.5°—1° C.) generally immediately, in the diminution of the expectoration, and the clearing of the physical edges. Scheffler prescribes it in a mixture thus:

| | | |
|---|---|---|
| ℞ | Camphor ... ... ... ... ... | gr. ¾ |
| | Tr. Quillaiæ ... ... ... ... ... | ♏ 30 |
| | Glycerin ... ... ... ... ... | ♏ 30 |
| | Syr. Tolu ... ... ... ... ... | ʒj. |
| | Syr. aurant ... ... ... ... ... | ʒss. |
| | Aq. ad ... ... ... ... ... | ℥j. |

Codeia or other drugs may replace the syrup, according to the indications. The drug is rapidly eliminated, and so should be given frequently in small doses. Its action is possibly antiseptic as regards the lungs, and also stimulant to the nervous system. Volland ("Therap. Monartsh.," February 1906) has given a one in ten solution of camphor in oil subcutaneously in pulmonary phthisis. Daily injections were given with a Pravatz syringe; sometimes as much as four syringefuls were given daily. Small doses are said to be useless, but with efficient dosage night sweats disappear and the heart's action is strengthened.

Claret ("Presse Méd., November 1, 1905) recommends the following solution for hypodermic use:

| | | |
|---|---|---|
| ℞ | Caffeinæ ... ... ... ... ... ... | gr. 4 |
| | Sodii salicylatis ... ... ... ... ... | gr. 4 |
| | Aquam ad ... ... ... ... ... | ♏ 15 |

Mix thoroughly and add—

| | |
|---|---|
| Sp. camph. 10 per cent. ... ... ... | ♏ 15 |

This solution is said to remain perfectly clear for a considerable time. It could be employed as a cardiac stimulant.

Gillette ("Am. Journ. Surg.," December 1905) advocates the external use of camphor carbolic acid in a variety of conditions, and gives certain practical rules for the preparation of the compound. Three parts of camphor to one of carbolic acid crystals are rubbed up with gentle heat. Glycerine must not be used, as the mixture is then but imperfect. This prepartion is permanent, and may be used as a dressing for wounds, either painted on or applied on gauze or wool. It does not produce a white eschar like carbolic acid pure, and hence need not be washed with alcohol. It may be used for swabbing the tonsils in acute tonsillitis. It mixes in any proportion with liquid vaseline (or petrolatum U.S.P.), and thus may be placed in an atomizer for spraying the throat. Its application to open wounds is absolutely painless.

